# Supplementary material for: Assessment of the willingness of dentists in the state of Indiana to administer vaccines
Source: PLoS One. 2022 Apr 19;17(4):e0267167. doi: 10.1371/journal.pone.0267167 (PMC9017890; doi:10.1371/journal.pone.0267167)
Supplement: S1 File — (DOCX) [file pone.0267167.s001.docx]

Appendix I

1. In what type of setting do you practice? (Check the one setting in which you spend the most time)
2. Private Practice
3. Specialty Practice-(Specialty name----)
4. Hospital Based Clinic
5. Dental Service Organization (e.g., Aspen/Kool Smiles etc.)
6. Academic Institution
7. Local Health Department
8. Federally Qualified Health Center (FQHC)
9. Mobile Dentistry Practice
10. Non-Profit/Free Clinics (“not an FQHC”)
11. Other ____________________
12. In what zip code do you practice the most time? ____________________________
13. Is your practice located in a
    - 1. Rural area
      2. Urban area
14. How many years have you been practicing?
    1. 0-5 years
    2. 6-10 years
    3. 11-15 years
    4. 16-20 years
    5. 21 years or longer
    6. No longer practicing
15. Do you have a policy regarding oral cancer screening in your office?

Yes/No / I don’t know

1. Please rate your agreement with the following:

|  | **Strongly Disagree** | **Disagree** | **Neither agree nor disagree** | **Agree** | **Strongly agree** |
| --- | --- | --- | --- | --- | --- |
| There is scientific proof that immunization prevents infectious diseases |  |  |  |  |  |
| Everyone should be receiving the recommended vaccinations (excluding those with prohibiting medical conditions) |  |  |  |  |  |
| Given the COVID-19 pandemic, if the Indiana State Board of Dentistry authorizes dentists to provide vaccinations (emergency order, etc.), would you consider offering vaccination in your practice? |  |  |  |  |  |
| Dental providers are competent enough to be able to administer vaccines and need no further education/training |  |  |  |  |  |
| I am comfortable administering vaccines in children |  |  |  |  |  |
| I am comfortable administering vaccines in adults |  |  |  |  |  |
| Dentists should be allowed to administer HPV, Influenza, Hepatitis A and COVID 19 (when available) in children. |  |  |  |  |  |
| Dentists should be allowed to administer vaccines such as HPV, Influenza, Hepatitis A and COVID 19 (when available) in adults. |  |  |  |  |  |
| HPV related oropharyngeal cancers can be prevented by use of vaccines |  |  |  |  |  |
| It would be easier for patients to complete their HPV vaccine schedule if they were to receive it from their dentists |  |  |  |  |  |

1. Would you consider offering vaccinations in your practice, if allowed by legislation?

Yes/No

1. What are some of the challenges you may face in being able to administer vaccines? (Check all that apply)

- Insufficient Training/knowledge
- Comfort levels
- Role confusion
- Reimbursement
- Time
- Storage of vaccines/supply chain
- Other_____________________

Other comments regarding oral cancer screening, dentists’ knowledge, promotion and/or their role in administration of vaccines such as HPV, Influenza, Hepatitis A and COVID _________________________________ ______________________________________________________________
