# Supplementary material for: Assessment of the willingness of dentists in the state of Indiana to administer vaccines
Source: PLoS One. 2022 Apr 19;17(4):e0267167. doi: 10.1371/journal.pone.0267167 (PMC9017890; doi:10.1371/journal.pone.0267167)
Supplement: Attachment — Submitted filename: Final rebuttal 2.docx [file pone.0267167.s002.docx]

1/3/2022

Title: Assessment of the Readiness of Dentists in the State of Indiana to administer vaccines.

Dear Editors,

We would like to thank the reviewers for their valuable time and effort in reviewing our manuscript. We have addressed all the comments and made edits in the blinded manuscript suing track changes as recommended.

Please find our **responses** to each comment and suggestions below, for additional clarification.

I remain available for any questions.

Thanks again.

Dr. Anubhuti Shukla

**REVIEWER’S COMMENTS**

Abstract
“Dentists should be allowed to administer HPV, Influenza, Hep A and COVID 19” I think the word “vaccinations” might be missing from that sentence.

**Response: Thanks for catching that**. **The term “vaccines” has been added as suggested.**

Background
“the Public Readiness and Emergency Preparedness (PREP) Act” is this specific for the state of Indiana or is it at US level?

**Response:** **This is a national level emergency authorization**

<https://www.phe.gov/Preparedness/legal/prepact/Pages/default.aspx>

The authors are presenting a comprehensive overview of the vaccinations that are being provided by dentists in various US states. As this is an international journal, it might strengthen the argument to mention a few examples of other countries where dentists have been allowed to provide vaccinations e.g., the UK.

**Response: Thanks for your comment. We included a new paragraph in the introduction and reported on the global data on COVID-19 vaccinations and the role of the dental community. (Paragraph 1)**

“Oropharyngeal cancers have been on the rise in the past three decades due to persistent high-risk-type human papillomavirus (HPV) infections”. Whilst I agree with the first part of the statement, that oropharyngeal cancers have been on the rise in the past three decades, I believe that it might be difficult to attribute this to a singular cause e.g., increased prevalence of HPV infections. It might be worth considering also mentioning the increasing role of the wider risk factors e.g., alcohol and smoking.

**Response: Out study was conducted in the US where the increase in the incidence of oropharyngeal cancers is mainly attributed to HPV high risk infections. We modified the sentence to make this clearer.**

“Pre-COVID 19 pandemic averages of HPV vaccine completion rates in the US were low (54.2%)”. Does this include both boys and girls or girls only?

**Response:** **yes, it includes both boys and girls**

“Expanding dental providers scope of practice to include vaccination could provide similar results as with pharmacists, however such changes nationwide would call for legislative enabling.” Would there be anything else needed? E.g., professional indemnity/insurance, training?

**Response:** **yes, we agree that educational training and professional indemnity may be needed. We modified the sentence as suggested by the reviewer.**

Results
“More than half of the respondents (68%) reported having a policy for oral cancer screening in their office.” I couldn’t find this data in Table 1.

**Response**: **Since this was a question not directly related to vaccinations, it wasn’t included in the table (data not shown).**

I noticed that the authors have presented in several places p values to attribute statistical significance. I am wondering if this really necessary and if it adds value to the paper. A significant number of journals have been moving more towards “confidence intervals” instead of p values to inform readers about the precision of the results as a more robust measure than p values. Also, I noticed the number of participants in some of the subgroup analysis is quite small therefore some of the statistical significance might be due to chance. I am not a statistician, and I am happy to be challenged here but perhaps a sample size calculation might have been useful to avoid the risk of multiple testing bias. I think perhaps percentages and/or confidence intervals might be an alternative to consider by the authors.

**Response**: **Although confidence intervals can be a bit more robust than p-values for interpreting significance of results, adding confidence intervals to results from surveys such as this are difficult to present in a way that’s easy for the reader to follow – as an example, for each of the 50 percentages shown in Table 2 there would be an additional 2 percentages shown for each one if the confidence intervals are added – so it would have the 50 N’s, 50 calculated percentages, and 100 additional percentages representing the confidence intervals – for the first piece of Table 2, instead of ‘441 (78%)’, the table would show 441 (78%, 74%-81%). We feel that the tradeoff using the p-values to show the results concisely outweigh the additional information added by the confidence intervals.**

**Sample size calculations performed prior to conducting a survey are rarely informative. The actual response rates are unknown and expected response rates vary widely. Further, the calculations depend not only on the unknown expected response rate but also on the distributions of the responses to the individual questions (also unknown prior to data collection).**

The authors talk about “the level of agreement”. I am not sure if I understand this correctly. Is this level of agreement based on percentage of responses agreeing with a statement or based on the p values?

**Response:** **Thanks for the question. This was based on the number (%) of responses**

Table 3 and table 4 presents two columns: single variable and multivariable. The authors might wish to consider clarifying what are the variables.

**Response:** **Single-variable refers to statistical tests using only the specific characteristic. Multivariable refers to statistical tests that include all three characteristics (practice setting, years in practice, location of clinical practice) simultaneously in the same statistical model.**

Barriers (not referenced in tables).
Does the first sentence refer to all respondents in general or only dentists working in the public sector? The second sentence starts with “private practitioners”. Might be worth clarifying which group does the first sentence refer to.

**Response:** **We added the** **clarification as suggested.**

Discussion
The authors argue about the opportunities of using dentists for administering vaccinations. Whilst this is important and noteworthy, it might be worth considering that dental hygienists/therapists and trained dental nurses might also have a role, which might be even more cost effective. Not sure about the situation in the US but in some countries, flu vaccinations in GP surgeries are not always administered by GPs but by trained nurses. This frees up GP time to deal with day-to-day business that requires a qualified physician. Might be worth considering the implications of using the wider dental workforce, not just dentists, to administer vaccinations as long as they are properly trained, competent, indemnified and remunerated.

**Response:** **We completely agree that dental hygienists may play a role in vaccine administration. However, the role of mid-level providers in dentistry is very restrictive in the US. Therefore, while it makes complete sense, including dental hygienists and therapists will take much longer and a different advocacy effort.**

“More than half of our study respondents were private practitioners which is comparable to the practice distribution of dental providers in the state of Indiana, where most dentists (93.6%) work in private practices.” Does this sentence imply that the dentists working in public sector were over represented in the study?

**Response:** **Since the sampling method was random, we did not oversample any groups, the sentence simply states that the distribution of respondents is pretty similar to the dental practitioners’ distribution in Indiana**.

FQHCs and DSOs appear in multiple places. Might be worth repeating what these acronyms mean for a the non-US based readers.

**Response:** **they are both referenced on page 12, last paragraph.**

“Further research exploring non- traditional settings to overcome the infrastructure challenges of supply chain, storage and cost inefficiency may be helpful in improving participation of dental providers, especially in private practice settings.” Does cost inefficiency mean cost effectiveness in this context?

**Response: cost inefficiency refers to the vaccine storage investment needed vs the reimbursement for administering the vaccine to patients in the dental office.**

Thank you again for your helpful comments and suggestions. Please let us know if you have any additional questions.
