# Supplementary material for: Assessment of the willingness of dentists in the state of Indiana to administer vaccines
Source: PLoS One. 2022 Apr 19;17(4):e0267167. doi: 10.1371/journal.pone.0267167 (PMC9017890; doi:10.1371/journal.pone.0267167)
Supplement: Attachment — Submitted filename: rebuttal.docx [file pone.0267167.s003.docx]

2/9/2022

Title: Assessment of the Readiness of Dentists in the State of Indiana to administer vaccines.

Dear Editors,

We would like to thank the reviewers for their valuable time and effort in reviewing our manuscript. We have addressed all the comments and made edits in the blinded manuscript using track changes as recommended.

Please find our **responses** to each comment and suggestions below, for additional clarification.

I remain available for any questions.

Thanks again.

Dr. Anubhuti Shukla

**REVIEWER’S COMMENTS**

Reviewer #3: This study investigates a relevant question and answers it with details that can guide policymakers for vaccination efforts. I have some minor recommendations:

1. It would be helpful to give a rough estimate of how many vaccinators may be added to the state's vaccination workforce if dentists are allowed to vaccinate, based on the percentage of dentists in the study that said they would consider vaccinating if allowed. That calculation can be given in the form of the number of dentists that may join the vaccination workforce, as well as what percent that may increase the vaccination workforce. These values would have to be tempered with an admission that these are purely rough estimates based on willingness to consider vaccinating, rather than readiness to vaccinate.
**Response: Thanks for the suggestion. We made the suggested edits on page 13, 2^nd^ paragraph, lines 2-5.**

2. In the Introduction, 2nd paragraph, 2nd sentence, the statement "More than half of our study respondents were private practitioners which is comparable to the practice distribution of dental providers in the state of Indiana, where most dentists (93.6%) work in private practices" is hard to rectify. >50% and 93.6% seem to far apart to be considered comparable.
**Response: We modified the sentence and clarified our statement (please see page 13, 6-7^th^ line of the 2^nd^ paragraph)**

3. In a FQHC, which will likely already have medical personnel administering vaccines in the same building as a dental clinic, there may be limited value to having dentists administer vaccines. I recommend at least mentioning this, as well as any reason why it may still be beneficial to have dentists in FQHCs administering vaccines.
**Response: Thanks for bringing up this important point. We mentioned it as recommended on page 14, first paragraph.**

4. The authors reference a previous study that looked at pharmacists' willingness to vaccinate. Now, pharmacists are a major part of the COVID vaccination effort. It would be valuable to mention how well that study predicted the current stage of the COVID vaccination drive and pharmacists' involvement.
**Response: This is a great point. We expanded on this in Page 15, 3^rd^ paragraph**

5. Please explain what is meant by the term "social desirability" in page 18, top paragraph.

**Response: Social desirability bias is the tendency to underreport socially undesirable attitudes and behaviors and to over report more desirable attributes. The survey included questions around beliefs, attitudes, and comfort levels around vaccination by participants, which may be affected by this bias, especially about their role in participating in HPV vaccination efforts.**

6. Please check if more states now permit dentists to vaccinate, since the original submission of this manuscript.

**Response: We updated this section (please see page 4, last line)**
